# Effectiveness of architectural separation of septic and aseptic operating theatres for improving process quality and patient outcomes: a systematic review

**DOI:** 10.1186/s13643-018-0937-9

**Published:** 2019-01-09

**Authors:** Romy Scholz, Alexander Hönning, Julia Seifert, Nikolai Spranger, Dirk Stengel

**Affiliations:** 1 Centre for Clinical Research, BG Hospital Unfallkrankenhaus Berlin, Berlin, Germany; 2 Department of Trauma and Orthopaedic Surgery, BG Hospital Unfallkrankenhaus Berlin, Berlin, Germany; 3 Hospital Group of the Statutory Accident Insurance, Berlin, Germany

**Keywords:** Aseptic, Septic, Spatial separation, Operating room, Surgical site infection, Process quality

## Abstract

**Background:**

Architectural division of aseptic and septic operating theatres is a distinct structural feature of surgical departments in Germany. Internationally, hygienists and microbiologists mainly recommend functional separation (i.e. aseptic procedures first) without calling for separate operating floors and rooms. However, patients with severe musculoskeletal infections (e.g. joint empyema, spondylodiscitis, deep implant-associated infections) may benefit from the permanent availability of septic operating capacities without delay caused by an ongoing aseptic surgical program. A systematic literature review on the influence of a structural separation of septic and aseptic operating theatres on process and/or outcome quality has not yet been conducted.

**Methods:**

Systematic literature search in PubMed MEDLINE, Ovid Embase, CINAHL and the Cochrane Library, screening of referenced citations, and assessment of grey literature.

**Results:**

A total of 572 articles were found through the systematic literature search. No head-to-head studies (neither randomised, quasi-randomised nor observational) were identified which examined the impact of structural separation of septic and aseptic operating theatres on process and/or outcome quality.

**Conclusions:**

This review did not identify evidence in favour nor against architectural separation of septic or aseptic operating theatre. Specifically, there is no evidence of a harmful effect of architectural separation. Unless prospective studies, ideally randomised trials, will be available, it is unjustified to call for abolishing established hospital structures. Future investigations must address patient-centered endpoints, surgical site infections, process quality and hospital economy.

**Systematic review registration:**

PROSPERO (International prospective register of systematic reviews): CRD42018086568.

**Electronic supplementary material:**

The online version of this article (10.1186/s13643-018-0937-9) contains supplementary material, which is available to authorized users.

## Background

Architectural separation of aseptic and septic operating theatres is a traditional structural feature of many hospitals in Germany which may be less frequently found in other European countries and surgical departments around the globe.

The philosophy of dedicated, spatially separated septic operating theatres has been questioned by hospital hygienists and microbiologists. They argue the concept is outdated and inefficient, and that clean and contaminated operations may safely be carried out in the same suite given proper functional separation (i.e. ordering schedules from aseptic to septic procedures) and sufficient wipe disinfection.

In 2017, an exploratory analysis of 16 septic and 14 aseptic general surgical procedures performed in a single theatre without laminar air flow showed no differences in bacterial air contamination and sedimentation [1]. Based on the results, an accompanying editorial concluded that “separation of septic and aseptic surgical areas is obsolete” [2]. This interpretation is unjustified, as the study was not designed to compare different theatre environments in a head-to-head fashion.

Protagonists of structural separation stress its logistic advantages and potential benefits for patients with severe infections. Permanent availability of a septic theatre and surgical team may guarantee that often complex debridement and revision procedures are performed during regular working hours. Otherwise, according to the principle of functional separation, rather sick patients can only be operated on after the aseptic surgical program has been accomplished. Emergency cases demanding immediate intervention may further interrupt schedules and postpone planned septic surgeries.

Determining and improving process quality and patient outcomes in operating theatres emerged as an area of active research across the globe [3–29]. However, the influence of the architectural division of aseptic and septic operating theatres on process quality and patient outcomes remains unclear.

We conducted a systematic review addressing the question whether structural separation of septic and aseptic operating theatres compared to alternative room and floor architecture is associated with improved process and/or outcome quality.

## Methods

### Selection criteria and search strategy

This systematic review complied with PRISMA guidelines (see Additional file 1 for a completed checklist). The literature search was performed on March 9, 2018. We searched the following electronic bibliographic databases with no language or publication status limitations since January 1968: PubMed MEDLINE, Ovid Embase, CINAHL and the Cochrane Library. The PubMed MEDLINE search strategy can be found in Table 1. Publications were identified using a prospectively defined algorithm and pre-defined selection criteria. Additional literature (including grey literature) was identified by a snowball search (e.g. “related articles” in PubMed, reference lists of identified publications) and by an internet search on the websites of relevant institutions.

**Table 1:** PubMed MEDLINE search strategy (limited to publication date range 01.01.1968–present)

Search steps	
1. Procedure room* OR operating room* OR operation theatre* OR emergency theatre* OR orthopaedic theatre* [Title/Abstract]	
2. Hospital design and construction [MeSH]	
3. process quality OR transfer time* OR setup time* OR number of operations OR number of interventions OR efficiency OR turnover-time* OR on-time start* OR delay* OR regular hour* Or work flow	
4. 1 AND 2 AND 3	
5. Surgical Wound Infection [MeSH] OR Soft Tissue Infections [MeSH]	
6. septic* OR contamin* OR clean-contamin* OR aseptic* OR clean* OR steril* [Title/Abstract]	
7. 1 AND 5 AND 6	
8. 4 OR 7	

For the qualitative part of the systematic review, original papers and any other identified literature were considered. For the quantitative part, data from all types of comparative studies were potentially eligible for synthesis (i.e. randomised controlled trials [RCT], quasi-RCT and cohort studies), provided that a sufficient number of studies of adequate quality were available.

### Primary outcome

Influence of spatial separation of septic and aseptic operating rooms as a feature of structural quality on:

#### • Process quality

- Shortening of transfer and setup times

- Number of septic interventions performed during regular daily operating work hours, according to previous planning and schedules (e.g., 07:00 A.M. to 05:00 P.M.)

and

#### • Outcome quality

- Incidence of nosocomial infections (especially wound and soft tissue infections)

- Prevalence of multidrug-resistant pathogens such as methicillin-resistant *Staphylococcus aureus* spec. (MRSA), extended-spectrum beta-lactamase (ESBL)-producing bacteria, multidrug-resistant gram-negative bacteria (3/4 MRGN)

- Rate of healing in musculoskeletal and implant-associated infections

- Incidence of surgical and non-surgical complications

- Generic and disease-specific patient-centered outcomes

### Data extraction (selection and coding)

Records identified were exported to bibliographic software (EndNote V7.7, Thomson Reuters). Duplicate records were reviewed and excluded when necessary. Titles and abstracts of all identified articles were screened for eligibility against the review selection criteria. Screening of articles, data extraction and critical appraisal were independently undertaken by two reviewers, with a third reviewer consulted in case of disagreement. A list of potentially relevant studies read in full-text form but excluded from the review was provided, justifying exclusion of the particular citation from the review.

### Risk of bias (quality) assessment

The bias potential of included studies was evaluated using the ROBINS-I tool [30], which was developed for the assessment of quantitative non-randomised studies in systematic reviews. For this purpose, the risk of bias—low, moderate, severe or critical—was assessed by seven domains (confounding, selection of participants in the study, classification of interventions, deviations from intended interventions, missing data, measurement of results or interventions, selection of reported results) and then summarised for each study. Studies that were assessed in the overall assessment with a critical bias potential according to ROBINS-I were not used to answer the research question, as their results were considered to be too biased to contribute meaningful evidence.

### Strategy for data synthesis

Depending on the number of studies and the degree of heterogeneity, the best available evidence can be aggregated using a meta-analysis or narrative synthesis. In case of a sufficient number of studies, homogeneous data and a noncritical potential for bias, it was planned to apply a random-effects meta-analysis, taking into account variations in effect sizes between interventions and typically expanding confidence intervals in case of heterogeneity. We also considered a sensitivity analysis including only studies with low or medium risk of bias to test the robustness of primary results.

## Results

**Fig. 1:**
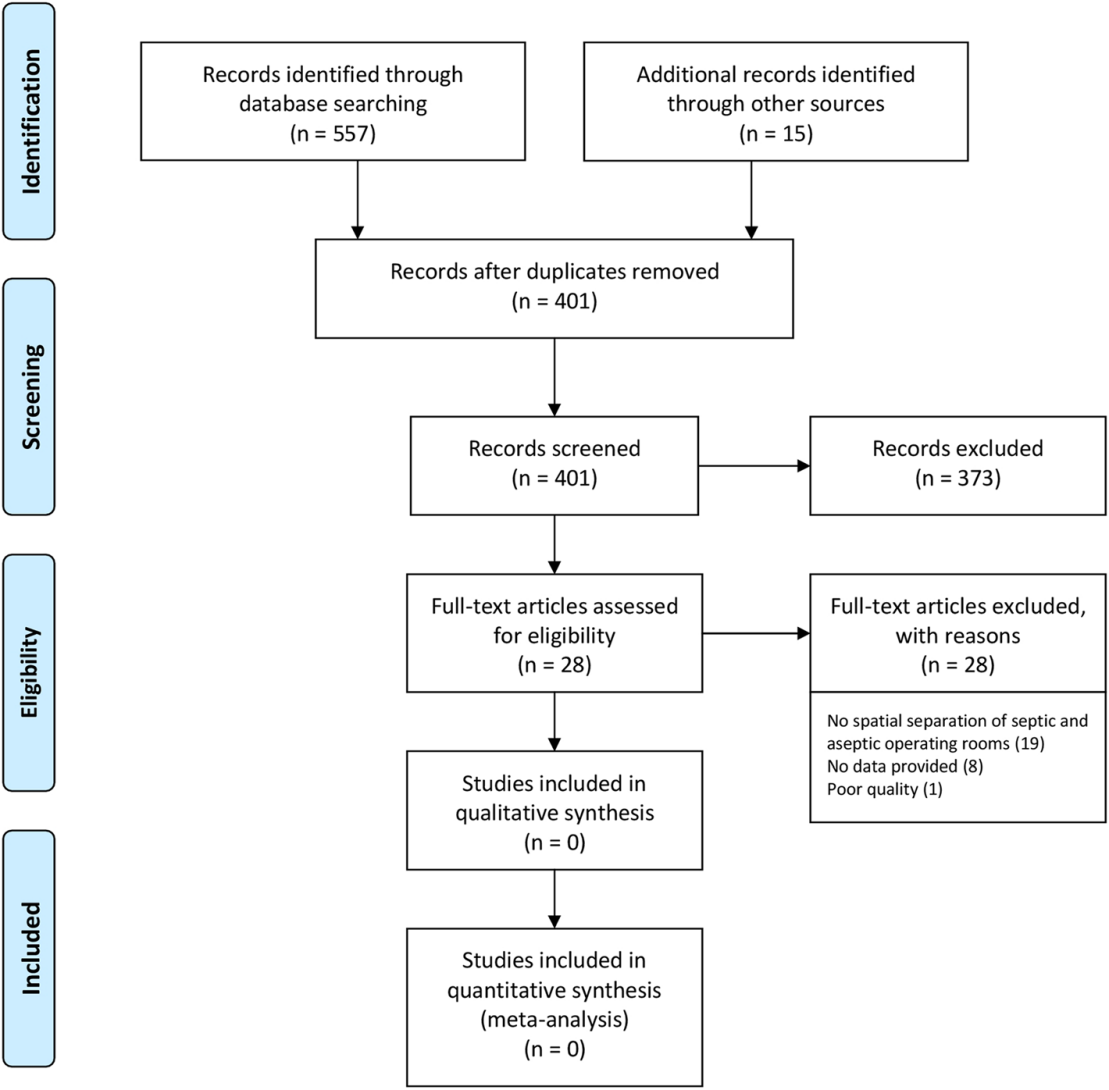
PRISMA flow chart

In the qualitative part of the review, a total of 572 articles were found through the systematic literature search in PubMed MEDLINE, Ovid Embase, CINAHL and the Cochrane Library, as well as through the identification of additional literature (Fig. 1).

Of these, 310 were on PubMed MEDLINE, 138 on Ovid Embase, 62 on CINAHL and 48 on the Cochrane Library. Fifteen articles were additionally identified through other sources. After the removal of duplicates, 401 articles remained, which were examined for suitability with regard to the selection criteria by title and abstract. Further, 373 articles were excluded because they could not be used to answer the research question. The remaining 28 articles were analysed in full text. Of these articles, 27 were excluded due to a lack of separation of septic or aseptic operating theatres or the articles did not contain evaluable data (correspondence, editorial).

Accordingly, only one investigation published more than 30 years ago remained which provided some information on the review question (Daschner et al. [31]).

Subject of the study by Daschner et al. was a qualitative and quantitative comparison of the bacterial spectrum of the air and floor of an aseptic and a septic surgery unit (Table 2).

**Table 2:** Study characteristics of Daschner et al.

	Septic operating theatre	Aseptic operating theatre
Objectives	To study the microbiology of air and floor samples obtained in an aseptic and septic operating theatre prior to (morning) and after completion of the daily surgical program (evening).	
Sampling method	One day per week during 5 months, before first surgery and after last surgery. Floor: RODAC blood agar plates at nine different points around the operating table. Air: Approximately middle of the room (25 cm higher than operating table) using a “Reuter-Centrifugal-Sampler” with a suction volume of 80.94 l in 2 min.	
Setting	Operating room, floor, gowning area for personnel only, washing room accessible through operating room only, all rooms except one for septic unit access were not closable/lockable, window ventilation	Clean air system, gowning area for patients and personnel
Disinfection method	Unit floor: 0.5% aldehyde disinfectant every day before first surgery and after last surgery, operating room floor after every single surgery	Unit floor: 0.5% aldehyde disinfectant every day after last surgery, operating room floor after every single surgery
No. of procedures	183	130
Air samples, mean CFU/m^3^ (SD)		
Morning	798.4 (552.4)	774.0 (826.2)
Evening	424.8 (301.1)	565.2 (521.0)
Plate samples, mean CFU/16 cm^2^		
Theatre, morning	9.3	6.4
Theatre, evening	31.5	8.4
Corridor, morning	18.8	12.8
Corridor, evening	14.8	18.3
Gowning area, morning	32.7	28.5
Gowning area, evening	53.4	56.1
SSI rate after 8 weeks	10.9%	3.0%

They stated that there was no significant difference in airborne bacteria and that floor contamination showed only little difference.

Wound infection rate was 10.9% for septic surgeries and 3% for aseptic surgeries. The responsible bacteria could not be isolated from the air or floor of the corresponding operating theatre. They suggest zoning of operating rooms rather than an architectural separation.

However, the publication had a critical risk of bias due to uncontrolled confounding (e.g. outdated disinfection measures, air conditioning of septic operating room by window ventilation). Thus, the study does not provide meaningful evidence to answer the research question.

## Discussion

The aim of the systematic literature analysis was to answer the question whether the structural separation of septic and aseptic operating rooms affects indicators of process and outcome quality. There are currently no head-to-head comparisons of structurally divided surgical floors with alternative approaches like functional separation of aseptic and septic procedures. The available indirect information is insufficient to draw meaningful conclusions on this problem either.

## Conclusions

Absence of evidence does not constitute evidence of absence. From a scientific point of view, it is currently unjustified to conclude that architectural separation is useless. The lack of convincing data in favour of this distinct structural feature should prompt prospective trials to study the impact of spatial segregation of septic and aseptic operating theatres on patient-centered endpoints (especially with regard to postoperative wound infections and soft tissue infections) and process quality.

## Additional file

Additional file 1: PRISMA 2009 Checklist. (DOC 62 kb)

## Abbreviations

CINAHL: Cumulative index to nursing and allied health literature
Embase: Excerpta medica database
MEDLINE: Medical literature analysis and retrieval system online
PRISMA: Preferred reporting items for systematic reviews and meta-analyses
ROBINS-I: A Cochrane risk of bias assessment tool: for non-randomised studies of interventions
SAV: Severe injury type procedure
VAV: Injury type procedure
